# Evaluation of the xenobiotic reaction against hyaluronate-based bioresorbable membrane in the abdominal cavity

**DOI:** 10.1007/s40204-016-0050-x

**Published:** 2016-07-04

**Authors:** Masaaki Nagata, Namiko Hoshi, Hayato Yoshinaka, Hideyuki Shiomi, Mamoru Takenaka, Atsuhiro Masuda, Yumi Maruyama, Ray Uchida, Takeshi Azuma, Hiromu Kutsumi

**Affiliations:** 1 Division of Gastroenterology, Kobe University Graduate School of Medicine, 7-5-2 Kusunoki-cho Chuo-ku, Kobe, Hyogo 650-0017 Japan; 2 Division of Radiation Oncology, Kobe University Graduate School of Medicine, 7-5-2 Kusunoki-cho Chuo-ku, Kobe, Hyogo 657-0017 Japan; 3 Center for Clinical Research and Advanced Medicine Establishment Shiga University of Medical Science, Seta Tsukinowa-cho, Otsu, Shiga 520-2192 Japan

**Keywords:** Foreign body reaction, Peritoneal adhesion, Seprafilm^®^, HBBM, Antiadhesive

## Abstract

Postoperative abdominal adhesions are one of the most common post-laparotomy complications observed. Several types of adhesion preventative agents are available and their effectiveness and adverse impact have been clinically evaluated in previous studies. However, few basic studies have tested whether those agents do not trigger any unwanted xenobiotic reaction, which makes some surgeons hesitant to use them. To clarify this point, we investigated whether the adhesion preventative agent Seprafilm^®^ (KAKEN PHARMACEUTICAL CO., LTD., Tokyo, Japan), one of the most widely used hyaluronate-based bioresorbable membrane (HBBM), can trigger an inflammatory response in normal abdominal tissue and delay the healing process. The rat underwent laparotomy and a HBBM was placed directly below the incision. Tissue samples at the incision and away from the incision (normal tissue) were harvested and inflammatory response and fibrosis were evaluated using quantitative PCR and histological scoring. We found that HBBM did not induce inflammatory cytokine expression at mRNA level in the peritoneal wall tissue or modify the fibrosis process in the abdominal cavity. These findings confirm the safety of using HBBM for the prevention of adhesion development post-laparotomy.

## Introduction

Postoperative abdominal adhesions are one of the most common complications observed post laparotomy (diZerega and Campeau 2001). These adhesions can cause many secondary problems including chronic abdominal pain, adhesive intestinal obstruction, and infertility (Stoica et al. 2014; Wang et al. 2009; McClain et al. 2011). Conservative treatment can improve the symptoms in many cases; however, additional surgical interventions, laparoscopy, or laparotomy are often required to lyse the adhesions and resolve the problems. Since abdominal adhesions occur in approximately 55–100 % of patients post general surgery (DeCherney and diZerega 1997), they not only reduce patients’ quality of life, but also constitute an economic burden on healthcare systems (Ray et al. 1993).

A number of postsurgical adhesion preventative agents are available. The efficacy of these agents has been evaluated in animal models (Bae et al. 2005; Lim et al. 2009; Ozcelik 2003; Harris et al. 1995; Shimizu et al. 2014; Oncel et al. 2005), as well as several large scale human clinical studies (Becker et al. 1996; Diamond 1996; González-Quintero and Cruz-Pachano 2009). The safety of these agents is usually determined by monitoring the patients’ vital signs and laboratory tests, which enables the detection of a systemic reaction against the preventative agents. However, xenobiotic reaction can happen locally (Böstman et al. 1990; Böstman 1992), and the effect of localized application of these reagents in the abdominal cavity, i.e., the potential adverse effects on normal tissues in the vicinity of the treatment site or remote tissues to which the reagent has spread, have not been examined.

A product called Seprafilm^®^ (KAKEN PHARMACEUTICAL CO., LTD., Tokyo, Japan), one of the most widely used hyaluronate-based bioresorbable membrane (HBBM), is a well-known adhesion preventative agent, however, little information about whether it can induce any unwanted immune reaction in the local tissue is not clarified. The aim of this study was to investigate whether inflammatory reactions, especially a xenobiotic reaction, are elicited by implanting a HBBM in the abdominal cavity. If no unfavorable xenobiotic reaction to normal tissue and no influence of healing process are confirmed, it should help to feel safe for the use of HBBM to reduce the post-laparotomy complications due to abdominal adhesions.

## Materials and methods

### Animal and operation procedures

Six-week-old male Sprague–Dawley rats were obtained from CREA Japan Inc, Tokyo, Japan. Rats were divided into four groups: Group (I) the Sham group that did not receive any intervention; Group (II) underwent laparotomy and visceral organ exposure (negative control group); Group (III) underwent laparotomy and visceral organ exposure and inflammation was then induced by scratching the ventral peritoneum (positive control group); and Group (IV) underwent laparotomy and visceral organs exposure and then hyaluronate-based bioresorbable membrane [HBBM; Seprafilm^®^, (KAKEN PHARMACEUTICAL CO., LTD., Tokyo, Japan)] was implanted (experimental group). All experimental protocols were approved by the Institutional Animal Care and Use Committees of Kobe University (Permission number: P140107) and conducted according to the Guidelines of Kobe University Animal Experimentation Regulations.

For the laparotomy, the rats were weighed and anesthetized in an induction chamber with isoflurane gas, and then placed on an insulated surgical table in ventrodorsal recumbency while anesthesia was maintained by isoflurane inhalation through an attached nose cone. Carprofen (Rimadyl, Zoetis Japan K.K., Tokyo, Japan) (0.05 mg/kg BW) was administered subcutaneously for analgesic purposes. The abdominal area was shaved and sterilized with iodine surgical scrubs and alcohol-soaked cotton balls. A vertical incision window approximately 2 cm in length was made with a surgical scalpel blade just caudal to the navel. A small perforation was made in the linea alba through the window, extended to 2 cm using a pair of scissors, and then the laparotomy procedure was completed. A simple continuous suturing pattern through both the peritoneum and the muscle layers was employed to close the inner incision with 5-0 PDS-II sutures (Johnson & Johnson K.K., Tokyo, Japan); 0.05 ml of Marcaine™ (Bupivacaine hydrochloride hydrate, AstraZeneca K.K., Osaka, Japan) was applied to the incision as a local analgesic aid. The skin was closed with a needle attached to 5-0 nylon suture (Monosof, COVIDIEN.CO, Tokyo, Japan) with a single interrupted pattern. Maintenance gas anesthesia was terminated following suturing and the rat was placed back in an individual cage following recovery. Rats in Group III underwent additional manipulation; the skin on both sides of the incision (5 mm from the center of the incision) was manually scratched with a sharp surgical curette until oozing was visually observed to ensure the occurrence of inflammation at the suturing site. The opening and closing procedures were the same for all groups. For Group IV, a 2 × 2 cm HBBM was placed on the greater omentum directly below the incision line prior to closing.

### Histology

Peritoneal tissue samples were collected 7 days following the operations. Samples were fixed with 10 % formalin and stained with hematoxylin–eosin (H&E) for microscopic observation. The samples were collected from three locations: the area of the peritoneum directly below the incision (all four groups), the area of the peritoneum 3 mm from the incision (where directly in contact with the HBBM in Group IV), and the area of the peritoneum 15 mm from the incision (where not adjacent to the HBBM in Group IV). For Group I, sampling locations were estimated since there were no incisions. The samples were evaluated in terms of the degree of inflammation and fibrosis and graded as follows. For inflammation: 0, no inflammation; 1, slight inflammation with a few lymphatic and plasma cells; 2, moderate inflammation with higher levels of lymphocytes, plasma cells, eosinophils, and neutrophils; and 3, severe inflammation with massive infiltration of inflammatory cells. For fibrosis: 0, no fibrosis; 1, low level fibrosis; 2, moderate fibrosis; and 3, severe fibrosis.

### RNA isolation and quantitative RT-PCR

Tissues samples collected from the peritoneum area directly below the incision were used. Tissue was homogenized in TRIZOL (Thermo Fisher Scientific Inc.) and total RNA was extracted according to manufacturer’s instructions. First strand cDNAs were prepared with Multi Scribe Reverse Transcriptase (Applied Biosystems) and qPCR was performed with SYBR Green reagents (Applied Biosystems) on a 7500 Real-Time PCR System (Applied Biosystems). The relative expression of the target genes was normalized to beta-actin expression.

### Statistical analysis

All evaluated data including the grade scores for inflammation and fibrosis and the expression levels of the inflammation markers determined by qPCR were analyzed with an unpaired two-tailed Student’s *t* test. Data were considered significant when *P* < 0.05 (Prism; GraphPad software, Inc.).

## Results

All rats exhibited good health and no weight loss during the entire course of the experiment, suggesting that neither the surgery nor HBBM implantation caused robust systemic adverse effects. At day 7 post operation, the HBBMs implanted in Group IV rats were almost completely dissolved and absorbed; small segments were apparent on the liver surface of 2 cases. By day 14, 28, 56, and 84, the HBBMs were completely dissolved and could no longer be detected (data not shown).

To investigate the possibility of a xenobiotic reaction against the HBBM, a microscopic examination was conducted to determine whether inflammation and fibrosis induction could be detected.

**Fig. 1:**
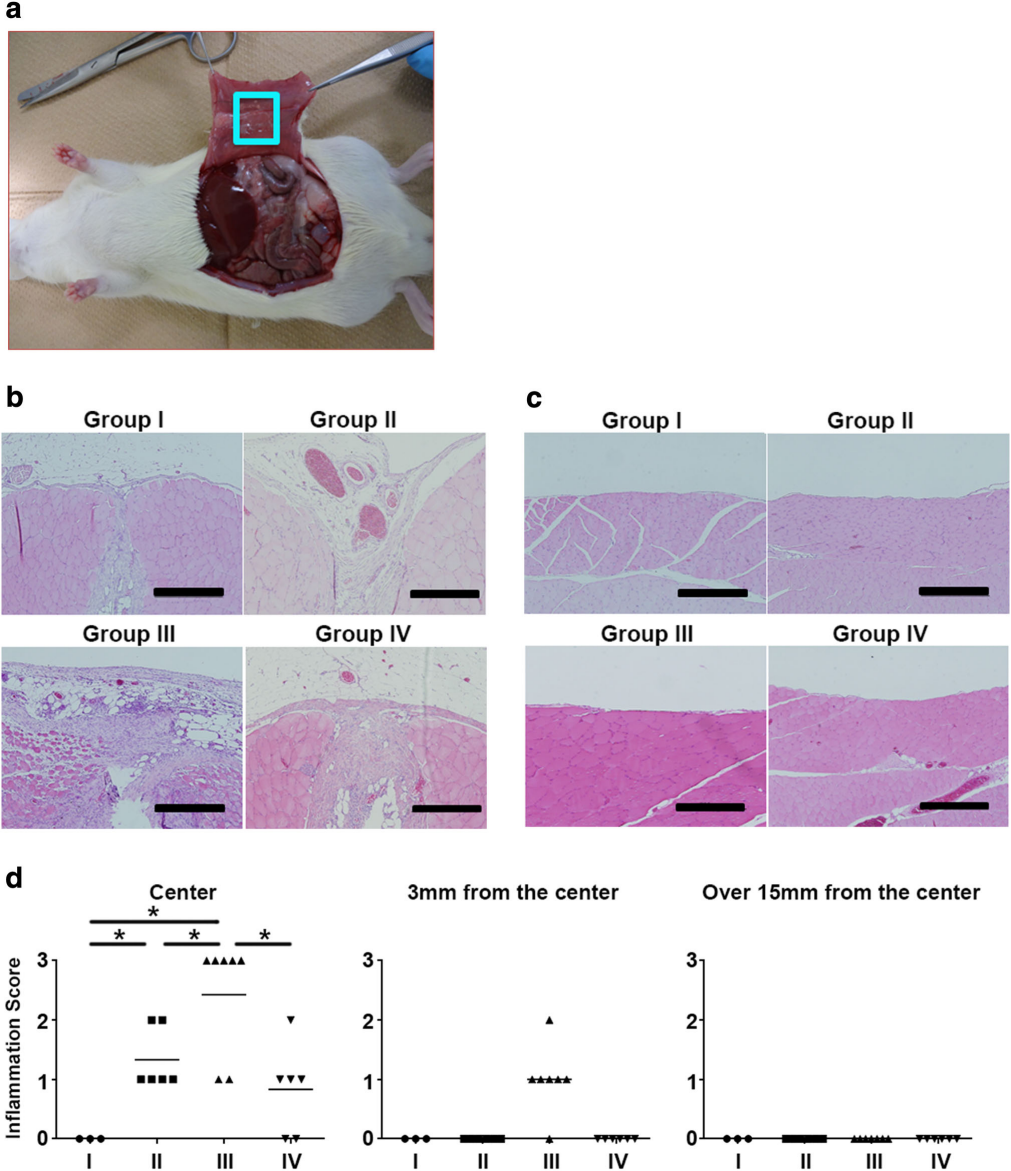
Histological evaluation of inflammation. **a** The image of rat operation. *Light blue line* indicates the location of sample excised. **b** H&E staining of tissue collected from the linea alba 7 days post operation. The *black bar* indicates a scale of 500 µm. **c** H& E staining of tissue 15 mm from the linea alba. The *black bar* indicates a scale of 500 µm. **d** Inflammation scores in H&E stained tissue collected 7 days post operation. *Left panel* linea alba; *middle panel* 3 mm from the linea alba; *right panel* 15 mm from the linea alba. Each *plot* indicates an individual rat. *P < 0.05, a significant difference (unpaired two-tailed Student’s *t* test)

Histological examination of samples collected from the peritoneum directly below the incision (Fig. 1a) did not reveal any inflammation (grade 0) in Group I (*n* = 3); four cases of grade 1 and two cases of grade 2 were observed in Group II (*n* = 6); two cases of grade 1 and 5 cases of grade 3 in Group III (*n* = 7, positive control for immunoreaction); and two cases of grade 0, three cases of grade 1, and one case of grade 2 in Group IV(*n* = 6) (Fig. 1b, d). These results confirm that there is no significant difference between groups II and IV in terms of the microscopic levels of inflammation. The tissue samples collected 3 and 15 mm from the incision were all evaluated as grade 0 with no apparent differences between the groups (Fig. 1c, d). These findings indicate that dissolved HBBM does not affect inflammation in the surrounding visceral organs.

**Fig. 2:**
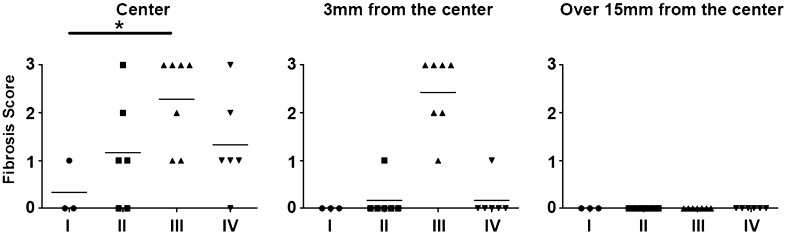
Histological evaluation of fibrosis. Inflammation scores of H&E stained tissue collected 7 days post operation. *Left panel*, linea alba; *middle panel* 3 mm from the linea alba; *right panel* 15 mm from the linea alba. Each *plot* indicates an individual rat. *P < 0.05, a significant difference (unpaired two-tailed Student’s *t* test)

To assess the contribution of HBBM to fibrillization or its effect on the fibrosis processes, the histological grading of fibrosis was evaluated (Figs. 1b, c, 2). In the samples collected from the peritoneum directly below the incision, there was two cases of grade 0 and one case of grade 1 in the Group I; two cases of grade 0, two cases of grade 1, one case of grade 2, and one case of grade 3 in Group II; two cases of grade 1, one case of grade 2, and four cases of grade 3 in Group III; and one case of grade 0, three cases of grade 1, one case of grade 2, and one case of grade 3 in the Group IV. These results suggest that there was no difference between groups II and IV and confirmed that HBBM has no clear modifying effects on fibrosis. None of the tissue samples harvested from other locations demonstrated any signs of fibrosis, indicating there were no adverse effects on the peritoneum in the vicinity of the incision.

**Fig. 3:**
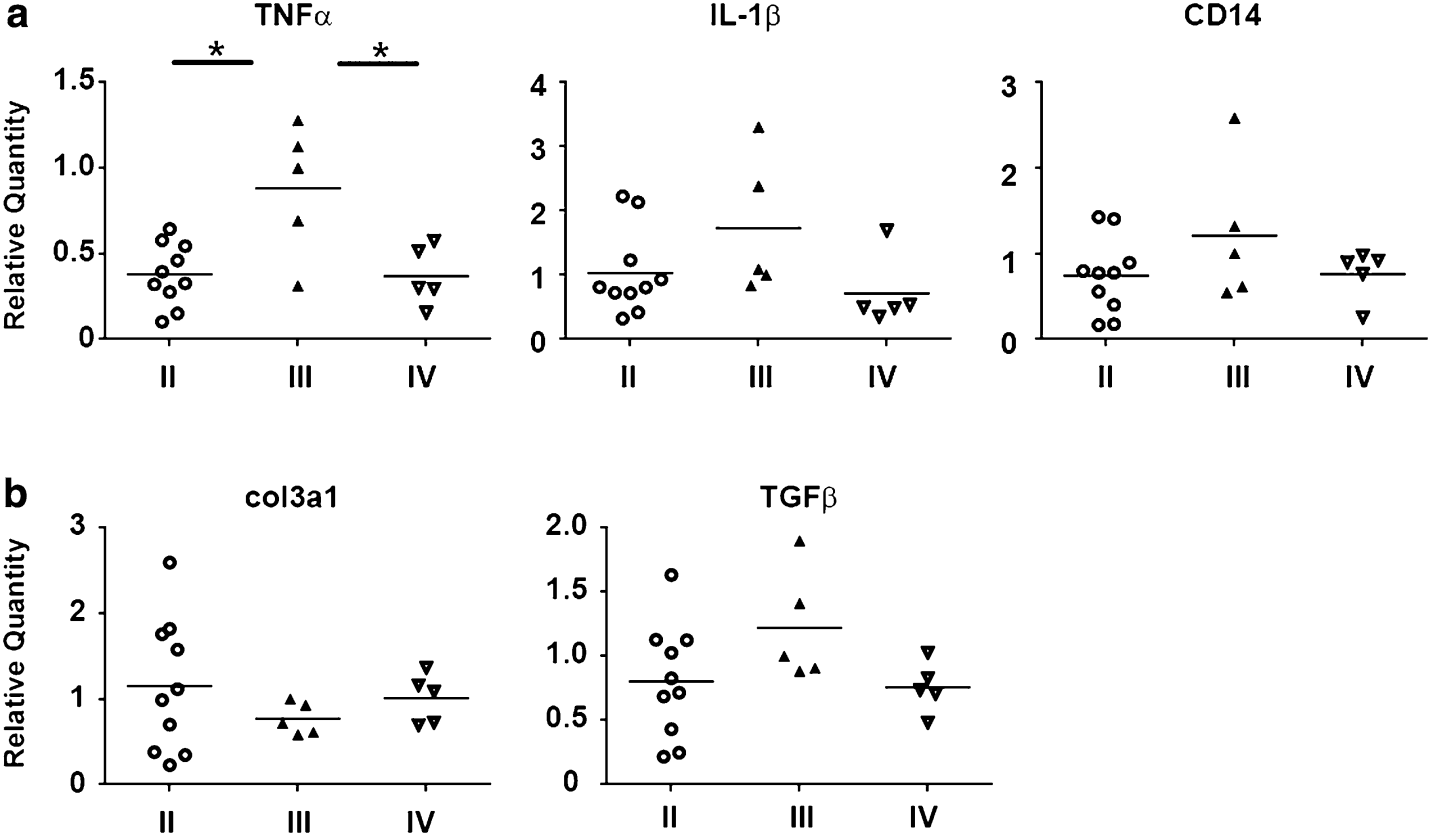
Gene expression levels of inflammatory markers. mRNA was isolated from linea alba peritoneal samples collected 7 days post operation. Expression levels were determined by qPCR. Each *plot* indicates an individual rat. *P < 0.05, a significant difference (unpaired two-tailed Student’s *t* test). **a** Evaluation of the inflammatory response: TNF-α, IL-1β, and monocyte/macrophage maker CD14. **b** Evaluation of fibrosis: col3a1 and TGF-β

Next, qPCR was utilized to detect the inflammatory reactions at the mRNA expression levels. Pro-inflammatory cytokines TNF-α and IL-1β and of CD14, an anchor protein found on monocytes and macrophages, which are inflammatory response cells, were tested. No apparent differences were observed between the levels of TNF-α, IL-1β, and CD14 in groups II and IV, further confirming the lack of an inflammatory response at the gene induction level or of cellular recruitment of monocyte and macrophage elicited by direct contact with the HBBM (Fig. 3a). Expression of the fibrosis markers col3a1 and TGF-β was also examined (Fig. 3b); no significant difference was apparent between groups II and IV, thus confirming that the HBBM does not lead to abnormal (delay nor unwanted acceleration of) fibrosis.

## Discussion

HBBM is mainly made of two components; hyaluronic acid and carboxymethylcellulose. Hyaluronic acid can be found throughout the body such in the connective tissue and the cartilage. Carboxymethylcellulose is widely used in medicine and synthetic compositions, and is known to be safe for the body. Therefore, one can expect that HBBM should be very safe as well. However, it requires testing it to confirm whether it is actually harmless without unexpected outcomes.

The rats were healthy during the experimental procedures and no differences were detected between the groups, indicating that the use of HBBM does not result in any obvious clinical problems. Adverse effects of HBBM reported in the clinical studies include the formation of intestinal obstructions and abdominal abscess (Becker et al. 1996; Diamond 1996); however, the occurrence of these was not seen in the current study. It suggests that those adverse effect happens only when surgical insults are involved, and not due to HBBM itself. In addition, our histology and qPCR analysis data showed that there was no apparent exacerbation or alleviation of inflammation at the site of incision following the application of the HBBM. Furthermore, the histology and qPCR results also indicated that HBBM does not have a significant effect on the fibrosis process.

HBBM works as a physical barrier between the tissues to keep them separated (Hooker et al. 1999). It can stay at where it was placed in the body for about one week to prevent from developing adhesions. There is a concern that the HBBM can dissolve and spread throughout the abdominal cavity thus affecting more remote normal tissue. Analysis of the tissue samples harvested 3 and 15 mm from the incision showed that application of the HBBM did not affect the normal tissue. This indicates that HBBM implantation does not induce any either microscopic or macroscopic adverse effects in the peritoneum regardless of whether there is direct or indirect contact with the HBBM. Taken together, it would seem that the HBBM is ignored by the host immune system and does not cause any xenobiotic reaction in the abdominal cavity.

This study revealed that implantation of the HBBM in the abdominal cavity does not induce a detectable inflammatory reaction at the microscopic level and does not modify the present inflammation or fibrosis process. The results of this study together with clinical reports demonstrating the effect of reducing post-surgical adhesion rates suggest that appropriate usage of HBBM may help to avoid developing abdominal adhesions and further secondary complications related to surgery as well as reducing medical expenses.

## Conclusions

HBBM induced no inflammatory response in the normal peritoneal tissue and did not modify level of inflammation nor the fibrosis development in the tissue after mechanical insult. Our data support the safety of HBBM and may help to encourage the use of HBBM to reduce post-laparotomy complications due to abdominal adhesions.
